# Policy entrepreneurs are integral in efforts to curb antimicrobial resistance in low and middle income countries

**DOI:** 10.3389/fpubh.2024.1292660

**Published:** 2024-03-12

**Authors:** Idemudia Imonikhe Otaigbe

**Affiliations:** Department of Medical Microbiology, School of Basic Clinical Sciences, Benjamin Carson (Snr) College of Health and Medical Sciences, Babcock University/Babcock University Teaching Hospital, Ilishan Remo, Ogun State, Nigeria

**Keywords:** antimicrobial resistance, policy entrepreneurs, policy windows, political will, health reforms, low and middle income countries

## Introduction

Antimicrobial resistance (AMR) is considered a global health challenge which results in an estimated 700,000 deaths annually (1). A failure to curb antimicrobial resistance (AMR) could result in a global catastrophe of 10 million deaths annually by 2050 (2). In low and middle-income countries (LMICs) the problem posed by AMR is having devastating consequences as AMR accounts for about 45% of deaths in Africa and South-East Asia. Also multidrug resistant (MDR) organisms such as extended spectrum beta lactamase producing *Klebsiella pneumoniae* have been associated with increased mortality in Africa and other parts of the world e.g., South East Asia, the Eastern Mediterranean and the Western Pacific (3). In addition an unmitigated rise in AMR and a paucity in research and development of new antimicrobials puts the world in grave danger of a post antibiotic era. In such an era, there would be an inability to treat minor infections due to a lack of effective antibiotics (3). Furthermore, the impact of a post-antibiotic era would be particularly severe in LMICs with high burdens of infectious diseases and weak health systems (3). It is therefore necessary to curb antimicrobial resistance in LMICs. Sadly, governments in many LMICs exhibit low political will to curb antimicrobial resistance (4–6). Clearly some LMICs have developed National Action Plans (NAPs) on AMR (7). However, low political will results in poor implementation of these NAPs (5, 8–10). Low politicàl will has also resulted in the suboptimal performance of other approaches which are fundamental in the fight against AMR. For example, many LMICs lack: adequate diagnostic microbiology services (11, 12); water sanitation and hygiene facilities (13–16); effective childhood vaccination services (17–19); access to effective antibiotics (20); infection prevention and control protocols in health care facilities (13–15); efficient surveillance structures (21–23); and reliable local data on antibiotic consumption and AMR (21–23). Certainly, other reasons (such as funding, lack of technical expertise, lack of multisectoral coordination, etc.) have been attributed to the poor efforts to curb AMR in LMICs (8–10). However, the low political will of LMIC governments is considered the most important factor hindering efforts to curb AMR (5, 6, 8). Political will is a term which refers to “the commitment of political leaders and bureaucrats to undertake actions to achieve a set of objectives and to sustain the costs of those actions over time” (24). The presence of political will creates a suitable environment to develop effective and sustainable regulatory frameworks to curb AMR (5, 6, 25, 26). Also when political will is present it fosters engagement and mutually beneficial partnerships between LMIC governments and the private sector (27–30). Such partnerships allow LMIC governments to leverage on key strengths of the private sector (e.g., technical expertise, capacity building, infrastructure, and financing) to curb AMR (31–40). It is therefore necessary to build political will to curb AMR in LMICs (5, 6, 8–10, 26, 29, 30, 40, 41). However, the complex socio-cultural, socio-economic and political dynamics in LMICs may make the process of building political will daunting and complex (42, 43). In this regard, studies have shown that individuals, often referred to as “policy entrepreneurs,” can be pivotal in building political will to implement health reforms (44–46). Therefore, policy entrepreneurs may play a pivotal role in tackling AMR in LMICs (6). The focus of this paper is to draw attention to the need to engage policy entrepreneurs in efforts to curb the menace of AMR in LMICs.

## Defining policy entrepreneurs

Policy entrepreneurs are individuals who actively engage (and collaborate) in efforts to promote reforms or innovations in national policy and decision making (45–48). Mintrom (46) describes policy entrepreneurs as “energetic actors who engage in collaborative efforts in and around government to promote policy innovations or health reforms.” Essentially policy entrepreneurs are skilled at introducing and promoting their ideas in many different fora (45–48). Also they invest time and energy to increase the chances for an idea to be placed on the decision agenda of the government (45–48). Policy entrepreneurs may be found anywhere in the sphere of policy and decision making (48). They may or may not be employed by the government or may hold elected appointed positions (48). They may be academics or individuals who work for advocacy groups or research institutions (48). Their willingness to commit and invest their resources (e.g., time, energy, reputation, finances, etc.) in the expectation of a future return is what clearly distinguishes them from other individuals involved in policy and decision making (48). They might receive that anticipated future return in the form of professional advancement, personal gratification, or the implementation of policies or regulations they are happy with (48).

In several LMICs, policy entrepreneurs have played useful roles in initiating and implementing health reforms (49–56). For example in Nigeria a health Minister successfully championed the implementation of Primary Health Care (49). Similarly in Uruguay, in 2007, the President supported by some politicians in government spearheaded health reforms which resulted in a National Integrated Health System (Sistema Nacional Integrado de Salud) designed to provide comprehensive and equitable health coverage for Uruguayans (53, 54). There are also examples of individuals outside government, civil society groups, and other non-governmental organizations which have been instrumental in building political will on burning issues (55, 56). For example in Kenya, a network of Civil Society Organizations used a combination of litigation and advocacy to ensure the revision of the 2008 Counterfeit Act, which had prohibited people living with HIV from accessing affordable generic drugs (55). Similarly in Indonesia, disability groups played a key role in efforts to pass legislation to protect the rights of people with disabilities (56). Also in China, a private enterprise served as a policy entrepreneur in the adoption of mobile healthcare payment (57). The success achieved by policy entrepreneurs in enabling these health reforms can also be adapted in efforts to curb AMR in LMICs (58).

## Policy entrepreneurs have a role to play in curbing antimicrobial resistance in low and middle income countries

The battle against AMR may be slow and frustrating if LMIC governments persistently display little or no political will to enact and enforce laws to curb antimicrobial resistance (58). However, a lot more can be achieved with the inclusion of policy entrepreneurs in efforts to curb AMR in LMICs (59). Several reasons support the preceding statement. Firstly, policy entrepreneurs understand the political dynamics involved in implementing health reforms (60). Therefore, the involvement of policy entrepreneurs in the reform process utilizes their political sagacity, enthusiasm and drive and provides the required momentum for policy adoption and diffusion (60). In addition, policy entrepreneurs can establish collaborative networks involving government, influential individuals, non-governmental institutions (who are involved in efforts to curb AMR in LMICs) and global organizations (60). Such networks can provide the required momentum to implement policies to curb AMR in LMICs (60).

Also, policy entrepreneurs are adept at creating or taking advantage of rare opportunities referred to as “policy windows” (48). Policy windows are described as “exceptional, fleeting periods of time when there is a greater likelihood of initiating policy change than usual” (61). For example the emergence of a Head of State or President with a passion to fight AMR is a significant policy window to curb the menace of AMR in a country (48). However, policy entrepreneurs are not expected to passively wait for policy windows to open or occur (62, 63). They can also proactively engage in activities which can lead to the creation or opening of policy windows regarding the issues of inappropriate antibiotic use and AMR (62, 63). Examples of these activities include: drawing attention to the dangers of inappropriate antibiotic use and AMR (e.g., through social media); building or strengthening coalitions with key stakeholders (e.g., influential politicians or citizens, research and policy organizations, etc.); and educating or increasing the knowledge of decision (or policy-makers) about inappropriate antibiotic use and AMR (58).

## Conclusion

Policy entrepreneurs have been instrumental in achieving health reforms in LMICs (49–56). They possess a keen understanding of the political process and are instrumental in building political will to implement health reforms (45–48, 60). Notably, a paucity of political will is a major factor impeding the fight against AMR in many LMICs (5, 8–10). However, the involvement of policy entrepreneurs will be instrumental in building political will to tackle AMR in LMICs (58–60).

## Author contributions

IO: Conceptualization, Formal analysis, Methodology, Supervision, Validation, Visualization, Writing—original draft, Writing—review & editing.
